# Effect of anemia on coagulation and platelet function: a whole blood *in vitro *study

**DOI:** 10.1186/cc9865

**Published:** 2011-03-11

**Authors:** G Scharbert, L Wetzel, L Berlinger, S Kozek-Langenecker

**Affiliations:** 1 Medical University Vienna, Austria; 2 Evangelical Hospital, Vienna, Austria

## Introduction

It is known that red blood cells are involved in hemostasis. They can support and improve coagulation in different ways. Therefore recommendations are given for red blood cell transfusions in anemic patients with massive bleeding to reach a hemoglobin concentration of 8 to 10 g/dl. Although blood transfusions can be life-saving, a number of negative or even potentially life-threatening effects are described.

## Methods

In this study we investigated the effect of anemia on platelet function and plasmatic hemostasis with two different point-of-care methods: the multiple electrode aggregometry Multiplate^® ^(MEA) and the rotational thrombelastometry ROTEM^®^. Blood was taken from 13 healthy volunteers to arrange *in vitro *anemia-series with 10, 7 and 3 g/dl hemoglobin. For the MEA we applied the agonists collagen, arachidonic acid, adenosine diphosphate (ADP), thrombin-receptor-activating peptide (TRAP) and ristocetin. For the ROTEM^® ^analysis we used the tests EXTEM, INTEM and FIBTEM.

## Results

The MEA showed significantly increased velocity of platelet aggregation in anemic blood samples. The agonists TRAP and ADP demonstrated the highest effects. The Aggregation Units and the area under the curve were not influenced by anemia. The ROTEM^® ^analysis displayed significantly an amplified maximum clot firmness (MCF), a shortened clot formation time (CFT) und an increased α-angle. The CFT and lysis index at 30 minutes did not show any changes through lowering hemoglobin. The calculated effect of platelets on ROTEM^® ^coagulation (MCF_platelet _= MCF_EXTEM _- MCF_FIBTEM_) was unchanged.

## Conclusions

In our study platelet function in anemic blood was observed with the MEA for the first time. Our results showed accelerated platelet aggregation through lowering hemoglobin. Our findings of a hypercoagulable profile in ROTEM^® ^are in accordance with earlier observations. Future clinical studies are needed to evaluate risk of bleeding or hypercoagulability in anemic patients.

